# A Polymer Optical Fiber Fuel Level Sensor: Application to Paramotoring and Powered Paragliding

**DOI:** 10.3390/s120506186

**Published:** 2012-05-10

**Authors:** David Sánchez Montero, Pedro Contreras Lallana, Carmen Vázquez

**Affiliations:** Electronics Technology Department, Carlos III de Madrid University, Avda. de la Universidad, 30, Leganés 28911, Madrid, Spain; E-Mails: pcontrer@ing.uc3m.es (P.C.L.); cvazquez@ing.uc3m.es (C.V.)

**Keywords:** fiber-optic sensor, intensity-based optical sensor, polymer optical fiber (POF)

## Abstract

A low-cost intensity-based polymer optical fiber (POF) sensor for fuel level measurements in paramotoring and powered paragliding is presented, exploiting the advantages of the optical fiber sensing technology. Experimental results demonstrate that the best option can be performed by stripping the fiber at the desired discrete points to measure the fuel level as well as with a gauge-shape fiber bending. The prototype has a good linearity, better than 4% full scale (F.S.), and sensitivity around 0.5 V per bend are obtained. Hysteresis due to residual fluid at the sensing points is found to be less than 9% F.S.

## 1. Introduction

Most of the basic principles and techniques for fiber-optic sensors (FOS) have been known for more than 40 years, but industrial applications are currently growing fostered by increasing diffusion of low-cost telecommunication components. Optical-fibre development to date has concentrated mostly on their use in telecommunications and data transfer systems, thus the principal stimulus for optical fibre sensors technology has been to provide a basic component set and also to facilitate specialist technologies through which slightly different versions of optical fibers can be fabricated purely for the sensing community. The number of fiber-optic sensors products can be expected to continue growing tremendously in the years to come as rapid progress continues to be made in the related optoelectronics and communications fields.

Fiber-optic sensors exhibit a set of very attractive characteristics, including immunity to electromagnetic interference, small-sized capability, resistance to hostile environments that may comprise hazardous chemicals or of any other kind, geometric versatility, ruggedness, sensor multiplexing and distributed sensing over a single fiber. On the other hand, their main disadvantages are sometimes their high cost (compared to other technologies), the unfamiliarity of the end user together with unexplored fields of application. There are numerous realizations of fiber-optic sensors but one extensively investigated transducing mechanism in optical sensing applications is the intensity modulation of the propagating light. Approaching simple configurations, intensity sensors modulate the optical power loss as the physical magnitude changes, thus providing the measurement as an optical intensity modulation signal thus facilitating their final commercialization and market spread. Intensity-based fiber-optic sensors have been demonstrated in literature to be very reliable, simple in concept, easily made selective to a specific measurand, easily integrated in optical networks by means of different multiplexing techniques, and a cost-effective sensing approach for a wide range of applications, and these are still increasing compared, for instance, to interferometric solutions [1].

On the other hand, industry frequently needs to measure fuel levels in tanks such as public-transport systems or service stations and any other large containers which implies exposure to harsh or highly flammable environments, as in the case of petroleum derivatives. Different methods such as mechanical, capacitive, inductive, ultrasonic [2], acoustic [3] or optical can be implemented. Typically, mechanical and ultrasonic methods are used to detect the level of solid materials that are in the form of dusts whereas capacitive and optical methods give better results in detecting fluid levels. Traditionally, in the case of gasoline stations, a common measurement method is to plunge a measuring rod into the underground tank to determine the fuel level. This rudimentary method tends to be slow and inefficient. In the automotive industry the fuel level is measured by a float connected to a variable resistance indicating the level of the liquid inside the tank. The main disadvantage of this system is that an electrical current must be introduced into the flammable (or simply conducting) liquid. In particular, if a flammable environment is a critical concern for industrial sensor applications, the optical solution is one of the best candidates to provide an intrinsically safe fuel level measurement scenario thanks to the passive nature of the light and dielectric properties of the fiber.

Fiber-optic sensor solutions have attractive properties for liquid-level measurements in practice. Moreover, submersion or flooding can be monitored by detecting radiation losses in bends and reflective intensity variations because of surrounding material changes. These applications can be carried out in oil tanks, containers, bio-mass boilers in condominiums, flood areas and underground [4,5], or even in lead-acid batteries [6]. At the same time, it should be outlined that in the optical sensing field, fiber-optic sensors can be constructed using polymer optical fibers (POFs) or silica-based versions, both singlemode (SMF) and multimode (MMF), but POFs have large numerical apertures, simple alignment to optical devices, high coupling efficiency, more flexibility, and lower cost. These are some reasons why new POF-based sensors have appeared and are still appearing, most of them based on optical power intensity detection. However, for remote monitoring over long distances, POFs have inherent disadvantages such as high attenuation losses and incompatibility with commercialized silica-based optical fibers.

In fact, optical fiber sensors for liquid level measurement have been extensively studied. Most liquid-level optical sensors are discrete or point-level sensors. In some cases, the sensor is a sensitive element which is submerged slightly in the liquid to indicate its presence, this element can be a non-fiber specific attached head such as a prism [7,8] or a reflective configuration based on fragile tilted fiber-optic [9], both based on total internal reflection (TIR). In those reflective configurations, multipoint measurements are based on using a new single receiving fiber for each measuring point, meaning a complex reception unit with wider area and weight [10]. Using these multiple receiving fibers, the multipoint approach is also used in some U bend configurations [6] or coupler configurations [11]. Other level sensors are based on serial U bend configurations but using cladding removed and core-polished bends, but these are more fragile configurations in the long term [12]. Some configurations are based on rod-type or U bend sections all along the same fiber, but with the jacket completely removed from the fiber [13], being less robust to undesirable wetting of liquids with high surface tension. Other sensors use fibers with clad and unclad zones [14] related to a specific code that are not easily automated for manufacture and they also need complex reception schemes, e.g., an optical decoding system, being difficult to develop portable devices in that case [15]. Intrusive schemes based on Fiber Bragg Grating (FBG) and Long Period Fiber Grating (LPG) sensors with mm resolution have also been reported [16], but they are fragile and non cost-effective technologies. On the other hand, optical solutions for liquid-level detection allowing continuous measurements have also been reported [17,18]. Nevertheless, in the solution proposed in [18] objective lenses need to be used as the light launched inside the tank tends to scatter and spread.

In this paper a U shaped bend configuration with only the jacket removed at discrete measuring points and flexibility to be placed on those non-symmetric points where the users need to know the liquid level will be proposed. A single fiber will be used to address and collect information for all sensing points [19].

**Figure 1.**
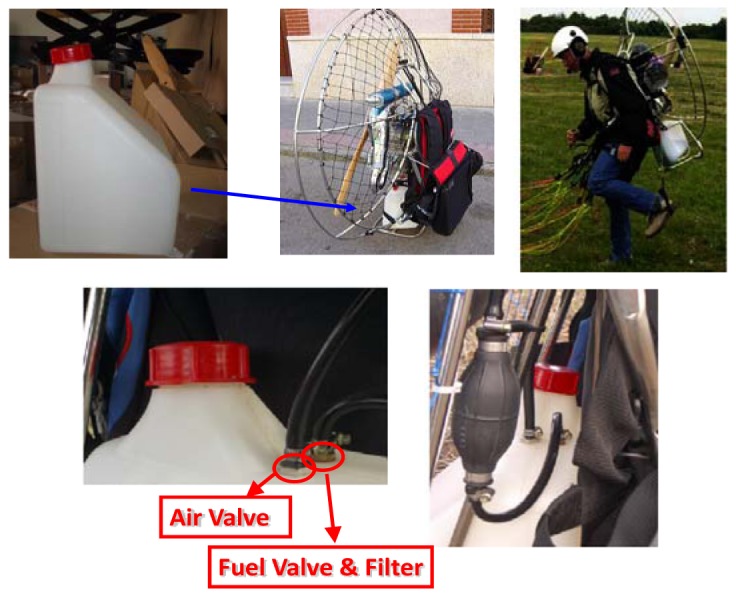
Real application scenario.

Paramotoring and powered paragliding are very recent sports which are seeing a growing demand for users' entertainment. Some pictures of the real application scenario are shown in Figure 1. A possible classification depending on basic features is given in Table 1. At the same time both sports are continuously increasing the demand of new and more sophisticated flight instrumental, but usually lack of real fuel level measurement, and estimations of the latter have to be done by relating the tank volume and the fuel consumption by mental arithmetic. Some exceptions can be found [20,21], but fuel probes work on the principle of changing capacitance and with specific housing to reach intrinsically safe sensors. Taking advantage of the aforementioned polymer optical fiber properties, in this work a compact POF intensity-based sensor for fuel level monitoring is developed thus providing a more significant safe flying scenario. The device provides all the advantages of POF sensors with tolerable impact in the total cost considering the last model that are available in the market. It is estimated a total overprice in the final product to the end users of less than 1%.

## 2. Sensor Description

The POF sensor described in this section is an intrinsic, quasi-distributed and intrusive fiber-optic measuring device for discrete-monitoring of fuel levels. The measuring points are distributed along the fiber length depending on specific requirements, and built on a cylindrical tube vertically positioned in a tank [19]. The principle of operation of the sensor is based on radiation losses in bends of optical fibers depending on the reflection coefficient at the boundary between two different media, at the measuring points where the fiber-optic sensor is located. The reflectance depends on the change in the refractive index of the surrounding medium. For the same incident angle the reflection coefficient is decreasing when the measuring point is immersed into a liquid having a higher refractive index. Finally, this reflection coefficient tends to zero as the refractive index of the surrounding medium approaches the fiber core refractive index. This implies that the received power at the output fiber end tends to decrease considering air (no-liquid), water or fuel, respectively.

For manufacturing the sensor, a low-cost commercial step-index PMMA (polymethylmethacrylate) POF of 980/1,000/2,200 μm core/cladding/jacket diameters, respectively, was used, with core and cladding refractive indices of *n_co_* = 1.492 and *n_cl_* = 1.417, respectively, and numerical aperture of 0.47. Restrictions imposed by the fuel tanks (typically up to 12 litres of fuel capacity and with specific shapes and access valves, see Figure 1 and Table 1) used in this industrial sector led us to evaluate different sensing prototypes vertically positioned inside the tank in order to test the most suitable option for this application, such as the type of bending (non-bend, twist- or gauge-shape). These different probes were prepared in order to investigate the sensing capabilities for several interfaces, *i.e.*, air, water and fuel, although being the latter the fluid of interest. It should be noted that refractive indices of these fluids are *n_air_* = 1, *n_water_* = 1.33, and *n_fuel_* = 1.42, respectively.

**Figure 2.**
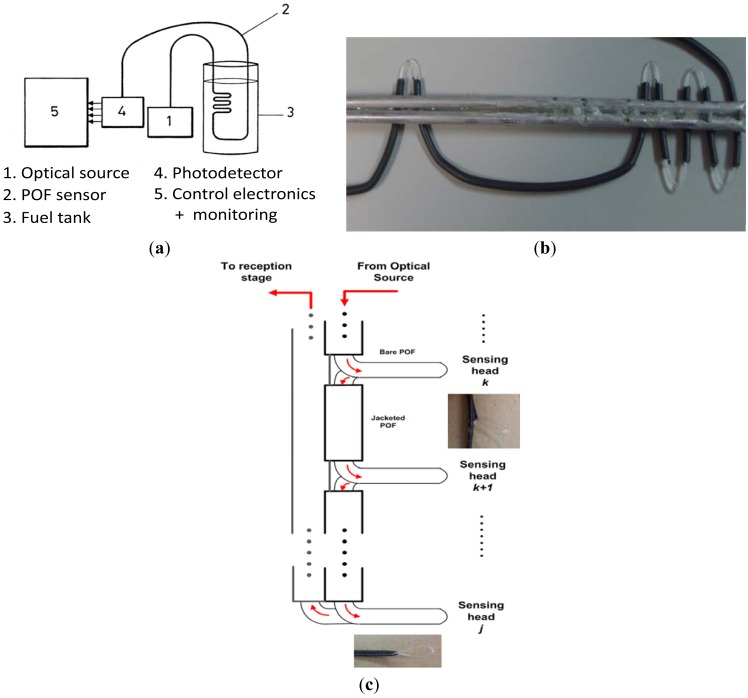
(**a**) Block diagram of the fuel level measuring system; (**b**) Photograph of a gauge-shaped POF-based fiber-optic sensor prototype for fuel level measurements; (**c**) Block diagram of the twist-shape POF fiber-optic sensor prototype.

The block diagram of the liquid level detection sensor is illustrated in Figure 2(a). A low-cost LED (IF-E96) operating at λ = 650 nm was employed to launch optical power to the POF-based sensing probe. At the reception stage a photodiode (IF-D91) was used thus obtaining an output voltage signal directly related to the level of the fluid in combination with an optoelectronic unit working as a liquid-level transducer. Finally an array of warning LEDs was implemented for full-time visual inspection by pilots. Commercial discrete electronic components were used for this control and monitoring stage.

The gauge-shaped sensing probe, illustrated in Figure 2(b), was fixed to a metallic stick providing more robustness to the sensor, with bending radius ∼15 mm and the jacket only removed at the measuring points. This bending radius comes from a trade-off between sensitivity of the sensor and radiation loss. As the bending radius decreases the detected optical power also decreases. Moreover, the high losses due to tight bends would reduce the number of measuring points that could be employed in the same fiber, for the proposed multilevel fuel level sensor. From ray tracing simulations [22], it is observed that in a POF fiber with a cladding thickness of 10 μm and a 9 mm bend radius, 4 dB radiation losses are obtained. In this design bend radius is increased up to 15 mm for having less losses per bend, allowing more measuring points in the same output full range. Figure 2(c) depicts a block diagram of the twist-shaped POF fiber-optic sensor prototype as well as some photographs of the sensing heads.

## 3. Experimental Results and Discussion

**Figure 3.**
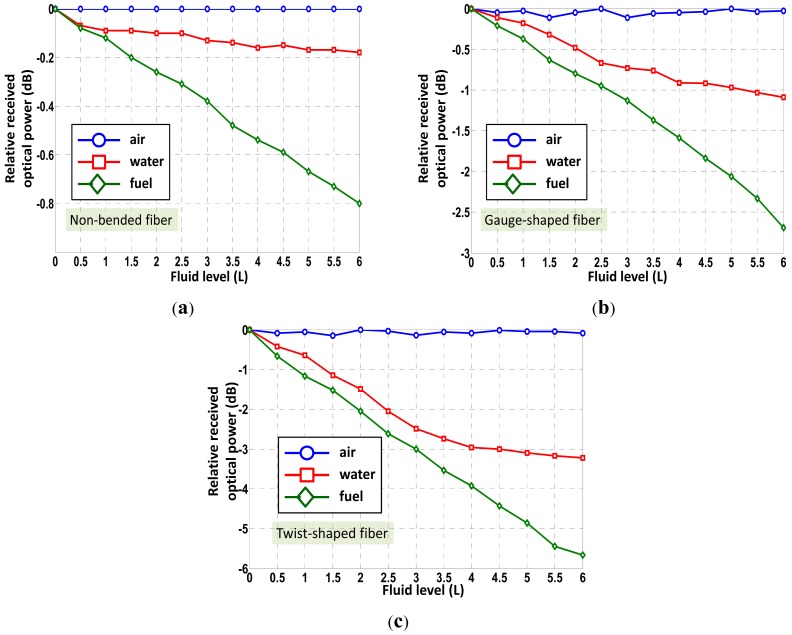
Relative optical power detected for different fluids and sensing probes as a function of the level of fluid. (**a**) Non-bended fiber sensing prototype; (**b**) Gauge-shaped fiber sensing prototype; (**c**) Twist-shaped fiber sensing prototype.

Experimental results are given in the following figures. A fuel tank of 6 L of capacity has been used for the experiments. The sensing points have been accordingly placed in height for measuring the fuel level in 0.5 L steps. This gives a total number of 12 sensing points within the fuel level full-scale (F.S.) analyzed. Figure 3(a) shows the relative optical power detected at reception *versus* fluid level (in L) for several sensing probes manufactured with different shapes. An average factor of Avg = 4 for each fluid at each fluid level condition has been applied. Time interval between each measurement was 10 seconds. As expected, as the refractive index of the external fluid increases and reaches values closer to that of the fiber (*n_co_* = 1.492), the reflection coefficient tends to zero and light is radiated outside the fiber. Consequently, less power is detected at reception. This fact can be seen comparing the experimental curves when the sensing probe is immersed into fuel compared to the other cases. Additionally, attending to the probe manufacturing best results in terms of sensitivity are obtained when applying a twist-shaped fiber (*i.e.*, full-turns of spire in the fiber). Linear regression coefficients concerning fuel level measurements were found to be r = 0.998 and r = 0.9928 for twist- and gauge-shaped sensing prototypes, respectively. It should be noted that no partial removal of core technique by polishing has been applied, although expecting a sensitivity enhancement [11] but being more fragile. The changes of the optical power signal detected at reception, proportional to the position and level of the liquid, were not a limiting factor for the application considered. Furthermore, standard deviation of measurements applying this technique tends to be higher. This fact can be seen as an error source dramatically limiting control electronics unit performance. For each probe curves of positive and negative fuel volume gradient were similar but did not overlap. In other words, the hysteresis of the sensing probes was small but observable. Nevertheless, it has been proposed for the prototypes to strip the fiber only at the desired measuring points thus its contribution can be minimized. The hysteresis was found to be less than 10% for all cases.

**Figure 4.**
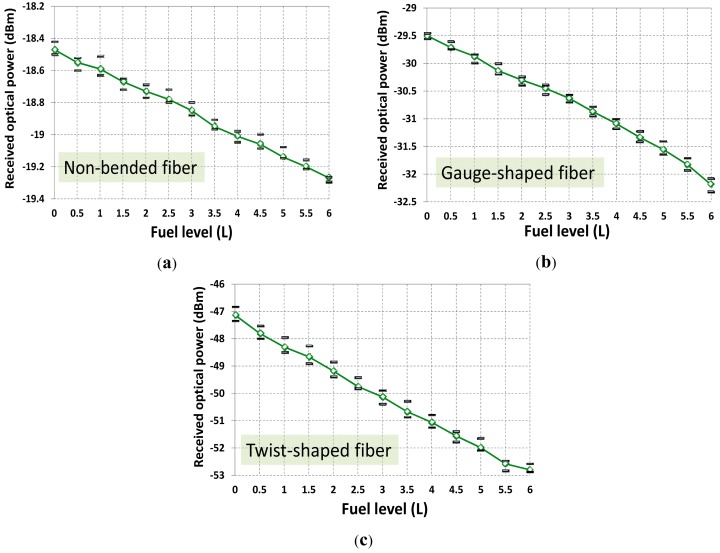
Optical power detected at reception *versus* fuel level for different sensing probes at 650 nm. (**a**) Non-bended fiber sensing prototype; (**b**) Gauge-shaped fiber sensing prototype; (**c**) Twist-shape fiber sensing prototype.

**Figure 5.**
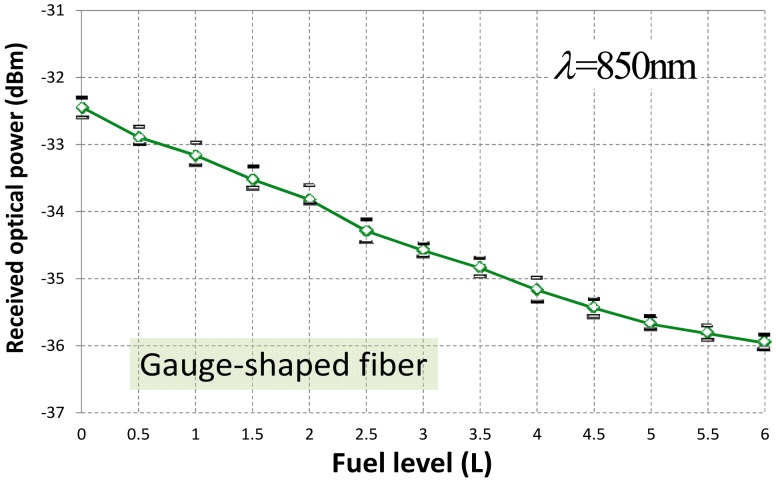
Optical power detected at reception *versus* fuel level for a gauge-shaped fiber sensing probe operating at 850 nm.

Focusing on experimental results on fuel level, Figure 4 shows the received optical power at reception stage for different sensing probes. Four measurements per fuel level have been carried out. From the experimental data, best sensitivity and highest linearity is obtained for a twist-shaped fiber configuration. On the contrary, less optical power is detected (this is due to the nature of the fiber bending shape) and larger standard deviation (variance) of measurements is achieved. Table 2 resumes all measurements statistics. Nevertheless the gauge-shape fiber sensing prototype was finally implemented. Key factor of decision was the low level of the optical signal detected for the case of a spire of fiber (i.e., twist-shaped). Nevertheless this fact could be easily overcome by launching more optical power (*i.e.*, using a high-power LED or laser diode) thus increasing the final deployment cost of this optical solution. Other solution could be to use a high-sensitive optical receiver but not leading to a cost-effective solution. And simplest and lowest cost solution as possible was considered to be the primary target for a final market spread. It should be noted that same set-up but employing a LED operating at λ = 850 nm was also tested. Same sensing probe operating conditions and averaging were also applied. Results are depicted in Figure 5 and summarized in Table 3. Despite the sensitivity enhancement, results showed a worse sensor linear response, and similar hysteresis was achieved. Results also showed that the optical power detected at reception decreased around a mean value of −3.9 dB despite the better photodiode performance at 850 nm. This is due to the dramatically higher POF losses at 850 nm [23], although POF length for the experiment was less than one meter. Nevertheless the limiting factor was the standard deviation (σ) which makes indistinguishable the fuel level analysis from 0.5 to 2 L, a range of prime importance in such scenario.

**Figure 6.**
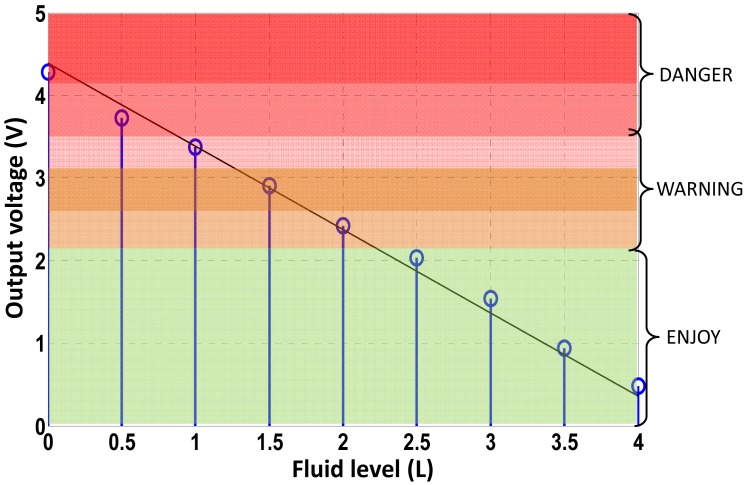
Calibration curve of the sensor prototype *versus* fuel level. Linear regression of the output voltage from the signal conditioning stage is represented in solid line.

From the above discussion, the best option is performed by simply stripping the fiber at the desired discrete multi-points with a gauge-shape bending, see Figure 2(b). Thanks to the POF flexibility the fiber is placed inside the tank through the input fuel valve. By means of a simple relation, and taking into account the tank geometry, the quantity of fuel (L) can be directly related to its height inside the tank, see Table 4. Consequently, at those points, the fiber was stripped and gauge-shape bended achieving the discrete points where the fuel level is being measured. Table 4 also shows the threshold voltage criteria implemented at the control electronics unit to discriminate the fuel level inside the tank. Level threshold criteria takes into account users' requirements, for instance, 2 L of fuel usually correspond to 40 minutes of remaining flight time, although depending on flight conditions. The calibration curve of the prototype *versus* fuel level is represented in Figure 6. Linear regression curve of the output voltage *versus* fuel level is also represented in solid line. Six warning areas have been configured, as users' request, and are illustrated within color regions of Figure 6. Nevertheless, scalability of the sensor system (both the POF-based prototype and the optoelectronics unit) would permit the increase of warning regions as desired. In addition, the control electronics stage has been designed to permit a recalibration of the threshold voltages shown in Table 3, due to the usage of different fluids, the usage of fuels with slightly different refractive indices, or undesirable power losses from the optical fiber system (e.g., thermal aging from the optical source), although a previous calibration performance should be required.

It should be mentioned that although temperature has been proven to change the optical power transmitted through the fiber, in the field of paramotoring and paragliding, fuel tanks are placed separately enough thus the motor heating source does not affect the fuel tank and, consequently, the sensor head. Minimum flight turbulences, horizontal courses and soft turns are the most usual flight conditions in this transport media and so variations in the fuel level measurements due to these circumstances can also be considered negligible in the performance of the sensor head. Anyway, in determined extreme cases (for instance, hard turns) the fuel could reach another height in which a sensing head is located. This fact can be considered as a residual fuel that remains on the surface of the fiber bend but from the experimental trials performed at laboratory, a recovery time of 5 minutes is estimated in order to return to the measurement of real fuel volume. Additionally, although extreme altitudes up to 5,400 m have been achieved, most flying recreational free-time activities are done below 150 m above ground level. This altitude corresponds to an environmental temperature decrease of less than 1 °C according to the International Standard Atmosphere (ISA). Although no real-field tests have been carried out, variations of the optical power detected due to this fact can be estimated to be negligible and adequately compensated for by the implemented control electronics.

## 4. Conclusions

In this paper, a POF-based fiber-optic sensor for fuel level discrete-monitoring applied to paramotoring and powered paragliding is presented. The optical sensing solution described performs an intrinsic, quasi-distributed and intrusive fiber-optic measuring device. The measuring points are distributed along the fiber length and built on a cylindrical tube vertically positioned in a tank. The principle of operation of the sensor is based on radiation losses in bends of optical fibers depending on the reflection coefficient at the boundary between two different media. The sensor system has been successfully demonstrated in the laboratory for three different surrounding media, and experimental results of three different prototypes are presented and discussed. A gauge-shaped fiber solution has been selected following previous discussion and calibration curve output voltage *versus* fuel level has been obtained, showing a non-linearity error of less than 4% F.S.; with sensitivities of 0.5 V per bend. Hysteresis due to residual fluid at the sensing points is found to be less than 9% F.S. This fuel level POF sensing solution is inexpensive with a very simple fabrication process, providing considerable cost savings from other fuel level sensors in the same field.

In addition, the control electronics, acting as a fuel-level transducer, provide a checking alternative to pilots by means of a hand-held visual electronic display compared to the traditional non-automatic visual inspections. Given that the optical fiber is the only part of the measuring device introduced into the tank- that is, only light and plastic interacts with the liquid- the method is safe, without electrical sparks that could cause a fire or a tank explosion.

## Figures and Tables

**Table 1.** Paramotoring classification and basic features.

**Range**	**Paramotoring basic features**					

	**Weight (kg)**	**Cylinder capacity (cc)**	**Power (HP/rpm)**	**Pilot max. weight (kg)**	**Propeler length (cm)**	**Fuel tank (L)**
Low-end	<23	<100	15/9,200	70	90	5 L
Mid-range	25	125	22/10,000	120	115	9–13.5 L
High-end	>26	>130	29/10,000	200	125	13.5 L

**Table 2.** Statistics of the experimental data in fuel level measurements at 650 nm.

	**Fuel level measurement**				

	**Linearity** ^a^	**Sensitivity**	**σ** ^b^	**Averaged optical power received** ^c^	**Hysteresis**
**No-bend**	0.9981	−0.07 dB/level	±0.05 dBm	−18.8 dBm	<11% F.S.
**Gauge**	0.9928	−0.22 dB/level	±0.13 dBm	−30.6 dBm	<8% F.S.
**Full-turn twist**	0.998	−0.47 dB/level	±0.38 dBm	−50.1 dBm	<9% F.S.

a Given by the linear regression coefficient, fuel level full-scale (F.S.);

b Maximum standard deviation in measurements, fuel level full-scale (F.S.);

c Measured at half-capacity, *i.e.*, 3 L.

**Table 3.** Statistics of the experimental data in fuel level measurement for gauge-shaped fiber sensing probe at 650 nm and 850 nm, respectively.

	**Fuel level measurement**				

	Linearity ^a^	Sensitivity	σ ^b^	Averaged optical power received ^c^	Hysteresis
**λ** = **650 nm**	0.9928	−0.22 dB/level	±0.13 dBm	−30.6 dBm	<8% F.S.
**λ** = **850 nm**	0.9751	−0.29 dB/level	±0.19 dBm	−34.5 dBm	<9% F.S.

a Given by the linear regression coefficient, fuel level full-scale (F.S.);

b Maximum standard deviation in measurements, fuel level full-scale (F.S.);

c Measured at half-capacity, *i.e.*, 3 L.

**Table 4.** Volume of fuel (in L), V(L), and corresponding heights (in cm), H(cm), inside the tank. Decision criteria (threshold voltage) implemented at the control electronics unit.

	**Fuel level fiber-optic intensity-based discrete-point sensor performance**					
**V(L)**	0.0	0.5	1.0	1.5	2.0	> 6.0
**H(cm)**	0.0	1.1	1.8	2.5	3.3	10.4
**Threshold (Volts), Vx**	Vx > 4.1V	4.1 > Vx > 3.5	3.5 > Vx > 3.1	3.1 > Vx > 2.7	2.7 > Vx > 2.2	2.2 > Vx
FL=Fuel Level (L)	FL = 0	0.5 > FL > 0	1.0 > FL > 0.5	1.5 > FL > 1.0	2.0 > FL > 1.5	FL > 6
